# OA06 Behçet's associated myositis: an unusual cause of exercise-induced leg pain

**DOI:** 10.1093/rap/rkad070.006

**Published:** 2023-09-27

**Authors:** Richard Porter, Pippa Watson, Eleftherios Agorogiannis

**Affiliations:** Manchester Foundation Trust, Manchester, United Kingdom; Manchester Foundation Trust, manchester, United Kingdom; Manchester Foundation Trust, Manchester, United Kingdom

## Abstract

**Introduction:**

This case describes a 29-year-old gentleman of Cypriot heritage who presented with exercise-induced bilateral lower leg pain and swelling which made it difficult for him to cycle. Symptoms progressed over months to calf pain at rest accompanied by fatigue. He subsequently presented with central visual loss in the right eye, followed by the left. The second episode of visual loss coincided with development of oro-genital ulceration and, following rheumatology review, a diagnosis of Behçet’s disease was made. The case is significant in terms of the chronicity and nature of presenting symptoms which is not typically described in this disease.

**Case description:**

A 29-year-old gentleman presented with exercise-induced bilateral lower leg pain and swelling for which he was reviewed in emergency care on four occasions. He received treatment for presumed cellulitis and underwent US-doppler studies, excluding thromboembolic disease. MRI of the lower legs was requested given lack of diagnosis with ongoing symptoms. This demonstrated bilateral muscle oedema in the antero-lateral compartments and lateral gastrocnemius heads, prompting rheumatology referral.

Prior to attending rheumatology, the patient developed bilateral sequential visual loss. This was attributed to right acute macular neuroretinopathy, followed by significant left posterior pole choroidal ischaemia 2 months later. Each presentation was associated with transient low-grade anterior uveitis, vitritis, and the presence of a pre-papillary vitreous infiltrate. Initial treatment included a six-week oral prednisolone taper from 60mg daily.

He reported a progressive leg pain which initially occurred during cycling but later affecting his walking. He was of Cypriot heritage, with no family history of autoimmune disease. A focused history revealed oral ulceration and an acne-like pustular rash over the back. His gait was antalgic with minimal toe-off phase. Investigations demonstrated raised ESR and CRP, negative autoimmune immunology, negative myositis ENA, normal CK, and positive HLA-B51. Given these features, a provisional diagnosis of Behçet’s was made.

Nerve conduction studies were requested to consider whether changes could be secondary to denervation. They displayed non-specific S1 neurogenic changes and small tibial motor amplitudes. Subsequent MRI lumbar spine excluded radiculopathy. Repeat CK remained within normal limits. Muscle biopsy was sought and performed with a clinical suspicion of atypical myositis which has previously been reported in Behçet’s.

At the second episode of visual loss, he was admitted under ophthalmology and treated with intravenous methylprednisolone and azathioprine. The case was discussed with our regional centre of excellence for Behçet’s and infliximab at 5mg/kg was commenced.

**Discussion:**

This case was notable for the complexity and atypical presenting features with calf pain induced by exercise likely due to atypical myositis (biopsy report still awaited at time of submission). More classical Behçet’s disease symptoms including oro-genital ulceration were significantly later. The case highlights the importance of multidisciplinary working and involving specialist centres especially when rapid escalation to biologic treatment is required. Leg symptoms are persistent but improved despite ongoing treatment with prednisolone and azathioprine with the recent addition of infliximab. Visual symptoms have stabilised and skin involvement has settled.

**Management Rationale:**

At the time of first review in rheumatology clinic, high-dose steroid treatment had already been initiated; therefore, expediting the investigative aspect of his management felt most appropriate, particularly given that myositis seemed to be the predominant feature at that time. In joint consultation with neurophysiology and neurology, putting the EMG and MRI findings in context, MRI of the lumbar spine was felt necessary to exclude discogenic disease causing denervation oedema. Unfortunately, visual symptoms progressed rapidly after this, requiring admission, which precluded an earlier opportunity for biopsy but leading to quick authorisation and organisation of treatment with infliximab.

**Key learning points:**

This case highlights an atypical presentation of Behçet’s disease including associated likely atypical myositis.

Causes of exercise-induced leg pain in a young, fit patient need to be considered:

The need for multidisciplinary discussion both across specialties and with regional centres is key in order to progress with diagnostics and treatment

In sharing this case for discussion, we wish to highlight possible causes of exercise-induced calf pain and a pathway for investigation of this. Our patient had atypical myositis as the cause for his symptoms in the context of multi-system disease. Both his myositis and ophthalmology presentations were somewhat atypical but multidisciplinary working, along with the development of some further symptoms, were helpful in making the diagnosis.

**Table:** OA06 Table

Differential	Clinical features	Investigation of choice
Chronic exertional compartment syndrome	Predictable onset pain and tightness within respective compartment. Resolves with rest	Intra-compartmental pressure measurement Post-exercise MRI
Bone stress injury	Gradual onset, localised pain. Reproducible with loading. Night/ rest pain if severe	MRI
Entrapment neuropathy	Paraesthesia associated with site +/- associated distal weakness *exclude referred lumbar radicular/stenosis	Nerve conduction studies MRI
Arterial endofibrosis (most commonly external iliac)	Associated with positions of hip flexion. Loss of power/endurance. Quick resolution on exercise cessation +/- bruit	Pre/post-exercise ABPI (<0.66 or deficit 0.18 significant)
Popliteal artery entrapment or aneurysm	Claudication symptoms and positional decrease in distal pulses. May be associated with tibial nerve symptoms	Dynamic Ultrasound with doppler +/- angiography
Tendinopathy	Initial stiffness which ‘warms-up’ with exercise initiation. Palpable pain/thickening localised to affected tendon, increased with resistance/stretching	Ultrasound Bloods to exclude metabolic/inflammatory cause
Thrombo-embolic disease and coagulopathy	Distal swelling, pain, erythema, paraesthesia	Ultrasound doppler, bloods

